# Autoimmune progesterone dermatitis: diagnostic relevance and epidemiological analysis of 13 cases

**DOI:** 10.61622/rbgo/2026rbgo30

**Published:** 2026-07-17

**Authors:** Nicole Touriel Bornsztein, Ariela Grabarz, Ana Laura Donaire Rapozero, Roberta Facchini Jardim Criado, Paulo Ricardo Criado, Gabriela Izzo Luiz, Luisa Homem de Mello Maciel Campilongo, Denise Maria Christofolini

**Affiliations:** 1 Centro Universitário Faculdade de Medicina do ABC Santo André SP Brazil Centro Universitário Faculdade de Medicina do ABC, Santo André, SP, Brazil; 2 Faculdade de Medicina do ABC Departamento de Dermatologia Santo André SP Brazil Departamento de Dermatologia da Faculdade de Medicina do ABC, Santo André, SP, Brazil; 3 Instituto Ideia Fértil de Saúde Reprodutiva Santo André SP Brazil Instituto Ideia Fértil de Saúde Reprodutiva, Santo André, SP, Brazil

**Keywords:** Autoimmune urticaria, Dermatitis, Progesterone, Menstrual cycle, Luteal phase

## Abstract

**Objective:**

Autoimmune progesterone dermatitis (APD) is a rare hypersensitivity to high levels of progesterone, with an as-yet unknown etiology. The objective of this study is to gather epidemiological data on patients with APD at an outpatient dermatology clinic.

**Methods:**

Medical records of patients diagnosed with APD, from menarche to menopause, seen at the dermatology clinic between 2004 and 2020 were reviewed.

**Results:**

Thirteen patients with APD were included. The mean age of symptom onset among APD patients was 24 years. The most prevalent clinical manifestation was urticaria, and the most common associated symptom was pruritus. The perimenstrual period was the most frequent aggravating factor, reported in 100% of cases. The intradermal progesterone test and autologous serum skin test were positive in approximately 60% of cases.

**Conclusion:**

Autoimmune progesterone dermatitis is an important differential diagnosis in female patients with dermatitis and should be considered during clinical evaluations.

## Introduction

Autoimmune progesterone dermatitis (APD), or progesterone hypersensitivity (PH), is a rare sensitivity to high levels of endogenous or exogenous progesterone.^(1)^ As it is a rare and still poorly recognized disorder, with only about 200 cases reported in the literature,^(2)^ its prevalence and incidence are not well established.

**Figure 1:**
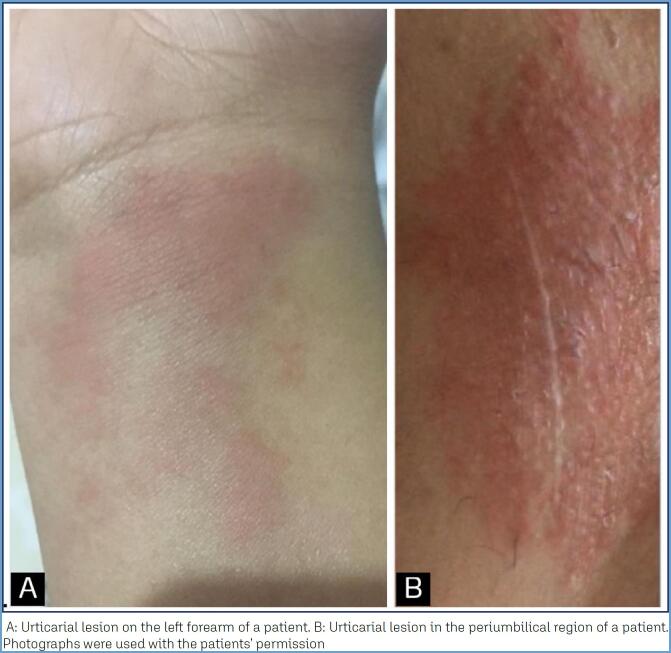
Examples of urticaria in two patients with APD.

APD encompasses a spectrum of dermatologic conditions induced by the menstrual cycle. The cutaneous manifestations of APD are diverse, ranging from urticaria to anaphylaxis.^(1)^ The clinical presentation may include eczema, erythema multiforme, fixed drug eruption-like lesions, folliculitis, stomatitis, papulovesicular eruptions, Stevens-Johnson Syndrome, and, most commonly, recurrent urticaria with or without angioedema.^(3)^ In more severe cases, patients may experience atypical anaphylactic reactions referred to as progesterone-induced anaphylaxis.^(3)^Figure 1 shows examples of urticaria in two patients with APD.

Symptoms generally occur 3 to 10 days before menstruation, at the peak of progesterone concentration, coinciding with the luteal phase of the menstrual cycle, and resolve at the onset of menstruation—hence, showing cyclical periodicity.^(1)^

The condition affects women of reproductive age and may be associated with endogenous progesterone during menarche or pregnancy,^(4)^ as well as exogenous sources such as contraceptive medications or in vitro fertilization therapies.

The diagnosis of APD is primarily clinical but may be confirmed through intradermal progesterone tests.^(3,5)^ A progesterone challenge test, whether intramuscular or oral, can also be performed.^(3)^ Additionally, skin prick tests and autologous serum skin tests may be used.^(6)^ Some patients may present with specific IgE to progesterone, detectable via ELISA.^(7)^

The primary aim of this study was to perform an epidemiological survey of patients presenting with dermatological manifestations related to autoimmune progesterone dermatitis. Secondary objectives included analyzing clinical findings—dermatologic and/or systemic manifestations, positivity for the autologous serum intradermal test, positivity for the intradermal progesterone test, and symptom correlation with pregnancy—in order to raise awareness of this underrecognized entity among healthcare professionals.

## Methods

A single-center, cross-sectional study was conducted involving patients from an outpatient dermatology clinic, diagnosed with APD between 2004 and 2020.

Inclusion criteria: Female patients from menarche to menopause who met the diagnostic criteria for APD and provided written informed consent. Given the absence of standardized diagnostic criteria for APD, the diagnosis was established based on an integration of diagnostic elements proposed by Buchheit and Bernstein^(8)^ and Chiarella et al.^(9)^ Specifically, patients were required to have a clinical history of skin lesions cyclically associated with the menstrual cycle (occurring 3-10 days before menses and resolving at its onset or shortly after) or in the setting of exogenous progesterone exposure, in addition to at least one of the following criteria: (i) improvement of symptoms after inhibition of ovulation or discontinuation of exogenous progestogen; (ii) a positive intradermal progesterone skin test; or (iii) a positive systemic progesterone challenge (oral or intravaginal administration).

Exclusion criteria: Patients who did not provide written informed consent, those with non-typical histories of cyclic dermatitis, and those who did not meet the diagnostic criteria for APD were excluded.

Data were extracted from the medical records of 13 patients with APD who met the inclusion and exclusion criteria. Collected variables included demographic data, clinical history and symptoms, positivity for the intradermal progesterone test and autologous serum test, response to treatment, symptom aggravating or alleviating factors, relationship with pregnancy, and age at symptom onset.

Patients underwent dermatological consultation, during which medical history was taken, along with general physical examination and comprehensive dermatologic assessment. The skin eruption pattern was characterized at the time of consultation when lesions were present, as some patients were in remission.

The following information was retrieved from patient records: current age, onset and duration of dermatosis, sex, symptoms associated with APD, factors that alleviated or worsened symptoms, positivity for the intradermal progesterone test and autologous serum test, whether a progesterone challenge test was performed, pregnancy history and symptom changes during pregnancy, history of angioedema or progesterone-induced anaphylaxis, whether monoclonal antibody treatment (omalizumab) was required, personal or family history of atopy, and use of hormonal contraceptives prior to dermatologic symptoms. When such data were absent from the medical records, patients were contacted directly during clinical visits or by phone to obtain the missing information.

The presence and results of the autologous serum skin test and the intradermal progesterone test were assessed, including the date performed, wheal diameter in millimeters, and test positivity.

**Figure 2:**
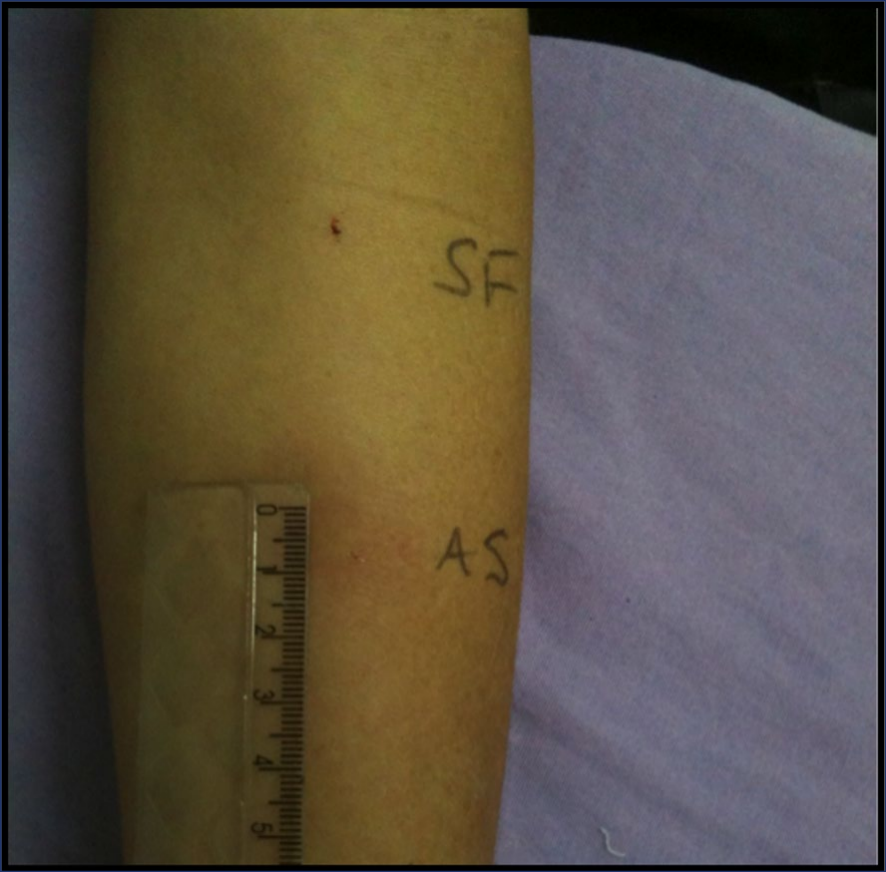
Positive intradermal test with autologous serum

The autologous serum skin test was performed as described by Sabroe et al.^(10)^ involving the collection of 5 mL of venous blood in a sterile tube without anticoagulant. The blood was left at room temperature for 30 minutes to allow clotting, then centrifuged for 15 minutes to separate the serum. After antisepsis with 70% alcohol, 0.05 mL of the patient's serum was injected intradermally into the volar surface of the right forearm using an insulin syringe. As a negative control, 0.05 mL of sterile saline was injected 5 cm away. On the left forearm, 0.01 mL of histamine (0.1%) was applied as a positive control. After 30 minutes, the horizontal and vertical diameters of each wheal were measured.^(10)^ The test was considered positive when the mean diameter of the serum-induced wheal exceeded that of saline by at least 1.5 mm, accompanied by erythema at the site of the autologous serum injection.^(10)^ To enhance specificity and sensitivity, the blood should be collected during a symptomatic phase of the disease (Figure 2).

**Figure 3:**
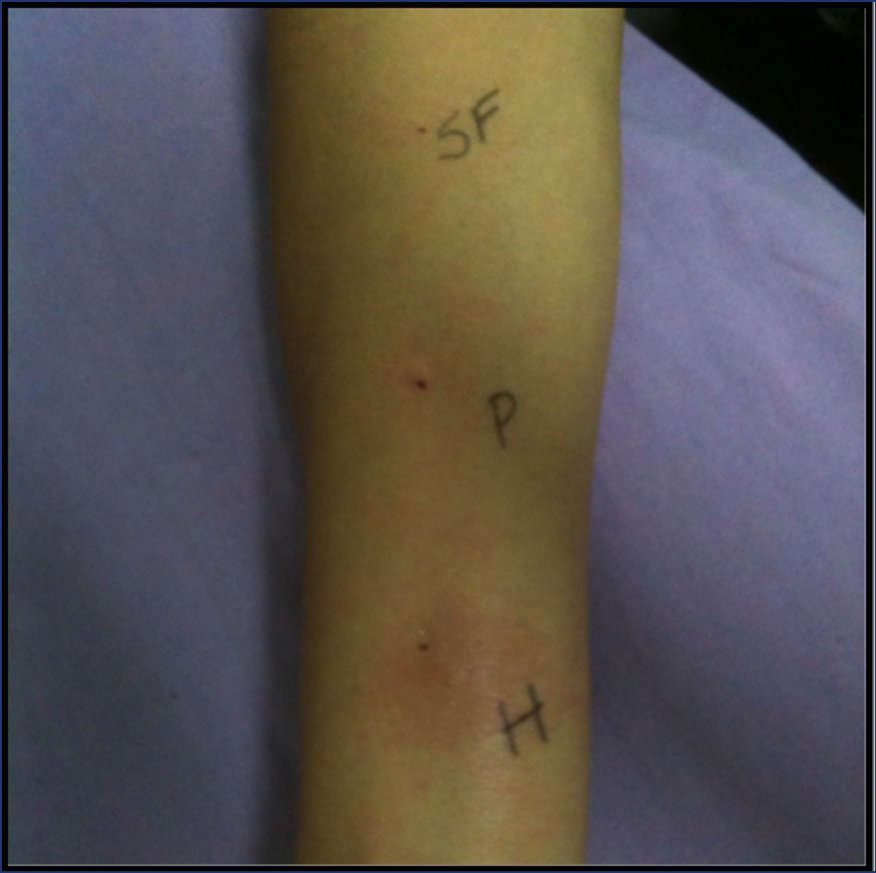
Positive intradermal test with progesterone

The intradermal progesterone test was performed as described by Mokhtari et al.,^(11)^ using aqueous medroxyprogesterone acetate solution at 0.1 mg/mL, administered intradermally, with results read at 20 minutes. Histamine served as the positive control and saline as the negative control (Figure 3).

Data collected from medical records were entered into an Excel spreadsheet and subsequently subjected to statistical analysis. Categorical variables (e.g., symptoms, alleviating and aggravating factors, medication use) were described using frequency and percentages. Continuous variables (e.g., age, age of symptom onset, histamine test wheal size in millimeters) were reported using mean and standard deviation for normally distributed data, or median and percentiles (p25–p75) for non-parametric data. The Shapiro-Wilk test was used to assess the normality of data distribution. Statistical significance was considered for p-values < 0.05, with a 95% confidence interval. All analyses were performed using R software, version 4.0.2.

Clinical data were obtained after presenting the study objectives and securing informed consent, as approved by the Ethics Committee 3.437.355 (CAAE 16198419.8.0000.0082).

## Results

Thirteen patients diagnosed with autoimmune progesterone dermatitis (APD) were included in the study. Participant information and test results are stated in table 1 and table 2. The mean age was 33 years, and the mean age of symptom onset was 24 years. The most prevalent symptom was pruritus, reported by all 13 patients (100%). Five patients (38%) presented with angioedema, and five (38%) experienced anaphylaxis. The most common morphological lesion was urticaria, observed in 9 patients (69%), followed by eczema and erythema multiforme. One patient (7%) presented with stomatitis. The most commonly reported alleviating factors were the use of antihistamines (11 patients, 84%) and corticosteroids (8 patients, 61%). Among the aggravating factors, the perimenstrual period was the most frequently reported, cited by 100% of the patients. Of the patients who had been pregnant, 4 (57%) reported worsening of their dermatitis during the prenatal and postnatal periods. Regarding diagnostic tests, the intradermal progesterone test was positive in 8 patients (61%), and the autologous serum skin test was positive in 5 out of 8 tested patients (62%). In total, 8 patients (61%) reported prior exposure to exogenous progesterone before the onset of clinical manifestations.

**Table 1:** Symptoms and test results

Variables	n(%)
Morphological types	
Urticaria	9(69.23)
Eczema	4(30.76)
Erythema multiforme	2(15.38)
Stomatitis	1(7.69)
Symptoms associated with APD	
Pruritus	13(100)
Angioedema	5(38.46)
Anaphylaxis	5(38.46)
Pain	4(30.76)
Burning	2(15.38)
Alleviating factors	13(100)
Antihistamines	11(84.61)
Corticosteroids	8(61.53)
Contraceptives	7(53.84)
Monoclonal antibody	5(38.46)
Cyclosporine	3(23.07)
Analgesics	1(7.69)
Aggravating factors	13(100)
Perimenstrual period	13(100)
Stress	3(23.07)
Pregnancy-related symptom changes	7(100)
Worsened during pregnancy	2(28.57)
Worsened postnatally	2(28.57)
Improved	1(14.28)
No change	2(28.57)
Progesterone test result	13(100)
Immediate positive	6(46.15)
Delayed positive	2(15.38)
Negative	4(30.76)
Negative with positive challenge test	1(7.69)
Autologous serum test	8(100)
Positive	5(62.5)
Negative	3(37.5)
Exposure to exogenous progesterone	
Before symptom onset	13(100)
Yes (exogenous)	8(61.53)
No (endogenous)	5(38.46)

**Table 2:** Age and histamine test results

Variable	Mean	Standard Deviation (min-max)
Age (years)	33.15	9.98 (17-54)
Age of symptom onset (years)	24.07	7.55 (12-36)
Histamine test result (mm)	4.75	1.21 (3-7)

Min: Minimum value; Max: Maximum value

## Discussion

Autoimmune progesterone dermatitis is a rare dermatologic condition that affects women during their reproductive years. In our study, we retrospectively analyzed 13 medical records of patients diagnosed with APD at an outpatient dermatology clinic—a significant sample considering that since 1921, when the first case of APD was reported, fewer than 200 cases have been described in the literature.^(2)^ The largest epidemiological study to date, conducted by Foer et al.,^(12)^ included 24 APD patients. This study represents one of the largest investigations on APD conducted in Latin America, offering a detailed analysis of the Brazilian epidemiological and clinical context. Additionally, it provides a critical perspective on the diagnostic methods currently employed, contributing to a more consistent understanding of the disease.

In our study, the majority of patients (69.23%) presented with chronic urticaria (with or without angioedema), 15.4% had eczema, and 15.4% presented with erythema multiforme, findings consistent with the literature.^(1,5,12,13)^ However, we found no cases of vesicobullous eruptions, which are also commonly reported.^(5)^ An extremely rare manifestation of APD, stomatitis, was the initial diagnosis in one of our patients (7.69%). This has been reported in only 1.12% of the 89 cases reviewed by Nguyen et al.^(5)^ and in none of the 24 cases analyzed by Foer et al.,^(12)^ or the 14 cases in Aghazadeh et al.^(13)^ Nguyen et al.^(5)^ reported angioedema associated with urticaria in 5.62% of cases. In contrast, over one-third of our patients (38.46%) experienced this manifestation, closer to the findings by Aghazadeh et al.,^(13)^ who observed it in 35.7% of cases.

The high frequency of angioedema in our cohort suggests a strong IgE-mediated allergic component, possibly with high histamine release. Several findings support this hypothesis: most of the positive intradermal progesterone tests were immediate-type reactions (IgE-mediated),^(5,14)^ and 84.61% of patients reported partial improvement with antihistamines, a proportion notably higher than reported in the literature.^(1,5,12)^ Additionally, 100% of our patients reported pruritus as a symptom of APD, which is associated with histaminergic pathways, while fewer than 50% reported this symptom in prior studies.^(5)^

Compared with prior case series, our cohort demonstrated a distinct clinical phenotype characterized by a higher prevalence of histaminergic manifestations. These findings suggest potential regional or immunological variability and support a predominantly Type I IgE-mediated hypersensitivity mechanism in this Brazilian population.

In Nguyen et al.^(5)^ study, only 50% of APD patients had prior exposure to exogenous progesterone, suggesting that oral contraceptives are not the sole triggers. In our sample, 61.53% had used exogenous progesterone prior to symptom onset, consistent with findings by Foer et al.^(12)^ (58%) and Aghazadeh et al.^(13)^ (64.3%).

Some researchers hypothesize that antigen-presenting cells may capture exogenous progesterone and stimulate helper T cells, leading to autoimmune sensitization.^(3)^ Conversely, cases without exogenous exposure may reflect low immune tolerance to endogenous progesterone.^(5,15)^ This variability in immune tolerance may explain differences in clinical presentation and response to treatment.^(5,15)^ Other sensitization mechanisms include cross-reactivity with hydrocortisone or related steroids,^(15)^ although no patients in our study had known steroid sensitivity. Hormonal cross-reactivity with antibodies to food or viral antigens has also been proposed.^(15)^

APD may develop or worsen during pregnancy, when progesterone levels are 10 to 5,000 times higher than in the non-pregnant state.^(2,15)^ In our cohort, only 7 of 13 patients had been pregnant. Among them, we identified three patterns of disease behavior: (i) 28.5% worsened during pregnancy, (ii) 57% had no change, and (iii) 14.5% improved.

Notably, half of the patients who reported no symptoms during pregnancy experienced worsening in the immediate postpartum period, suggesting sensitization to the high progesterone levels of pregnancy.^(12)^ One patient developed hypersensitivity only after her third delivery, highlighting that previous pregnancies do not exclude future intolerance to endogenous or exogenous progestogens.^(12)^

Paradoxically, one patient (14.5% of those who were pregnant) improved during pregnancy. Jenkins et al.^(16)^ proposed that gradual increases in progesterone may induce self-desensitization. This mechanism supports the use of desensitization therapy for patients who are refractory to standard treatments.^(2)^

The intradermal progesterone test may yield negative, immediate positive, or delayed positive results.^(8)^ A positive intradermal test strongly supports the diagnosis of APD; however, a negative result does not rule it out.^(8)^ In our study, five patients (38.46%) had a negative intradermal progesterone test. In these cases, it is proposed that progesterone may alter mast cell and/or basophil reactivity, directly activating them via hormonal induction and causing the release of bioactive mediators.^(17)^

Six patients (46.15%) had immediate positive intradermal tests, suggesting Type I IgE-mediated hypersensitivity reactions involving mast cells.^(5)^ Two others (15.38%) showed delayed positive results, indicating Type IV cell-mediated immune responses.^(5)^

Although the intradermal progesterone test is the most widely used diagnostic tool in APD, its accuracy remains questionable due to the lack of well-defined sensitivity and specificity, with some studies reporting values below satisfactory in these parameters.^(9)^ Additionally, lack of universal standardization regarding drug concentration and vehicle, along with reports of false-positive results, further limit its reliability.^(9,18)^ Consequently, these factors restrict its use as a standalone diagnostic test. In our cohort, negative results in a subset of patients reinforce that APD should remain primarily a clinical diagnosis, centered on the characteristic perimenstrual exacerbation observed in all patients.

In one patient with a negative intradermal test, a progesterone challenge test was conducted and was positive, confirming the diagnosis of APD. The remaining patients with negative test results did not undergo the challenge test due to its risks, since many presented with urticaria and angioedema, which could escalate to severe disease.^(13)^

The autologous serum skin test has been suggested as an useful tool in detecting cutaneous autoreactivity in certain autoimmune urticarias, such as those associated with anti-IgE autoimmunity.^(19)^ García-Ortega et al.^(6)^ proposed that this test may be valuable in indicating an autoimmune mechanism in APD. Nevertheless, the test has important limitations, as it does not distinguish between different autoimmune mechanisms and demonstrates limited specificity.^(19)^ In this study, eight patients underwent the test, which was positive in 62.5% of cases and negative in 37.5%, reinforcing its role as a supplementary, though not definitive diagnostic tool.

Other diagnostic tools include the progesterone skin prick test and the progesterone challenge test (oral or intravaginal), though these may worsen severe dermatitis and lead to prolonged morbidity.^(6,8,9)^ Patients with APD may also present with progesterone-specific IgE detected via ELISA, as demonstrated by Bernstein et al.,^(7)^ which may be the most practical and reliable method for confirming an IgE-mediated immune response.^(8)^

The mainstay of treatment is ovulation suppression using continuous oral contraceptives, which keep progesterone levels stable and prevent luteal phase peaks.^(1,12)^ Continuous use also prevents monthly resensitization that could occur if progestin is withdrawn to induce withdrawal bleeding.^(12)^ In our cohort, 54% of patients reported improvement with continuous contraceptive use, and two patients reported worsening when using the medication intermittently.

Antihistamines are generally the first-line treatment due to their safety profile. Topical corticosteroids may be used in milder cases, while systemic corticosteroids are reserved for exacerbations.^(5,20)^ However, long-term systemic steroid use is limited by adverse side effects.^(16)^ Although most studies report limited efficacy of these medications compared to ovulation suppression therapies,^(1)^ in this study, antihistamines followed by corticosteroids were the main therapies reported to provide symptom relief.

Some authors recommend gonadotropin-releasing hormone (GnRH) agonists in severe, treatment-resistant APD to suppress ovulation.^(21)^ However, use is limited to six months and may cause estrogen deficiency side effects, making this therapy more suitable for perimenopausal patients.^(22)^

Symptoms consistent with hypersensitivity have also been successfully treated with progesterone desensitization.^(12)^ Rapid desensitization protocols using intravaginal and intramuscular progesterone have been described, as well as slow oral desensitization protocols using continuous combined oral contraceptives.^(12)^ For patients seeking definitive symptom resolution, bilateral salpingo-oophorectomy with or without hysterectomy may be considered, though only in those with no desire for future fertility.^(5)^

With increasing use of oral contraceptives and supraphysiologic doses of progesterone in assisted reproduction, a higher prevalence of APD is expected in the future, highlighting the current relevance of this topic.^(5)^

Study limitations included the small number of recruited patients, due to the rarity of the condition, limiting statistical power; and the lack of a standardized serological gold standard test for APD to evaluate the sensitivity of intradermal testing.

## Conclusion

Various clinical presentations of APD were observed, encompassing a wide range of cutaneous manifestations. Nonetheless, chronic urticaria was the most common lesion in the studied group, with pruritus being the most frequently associated symptom. The intradermal progesterone test and the autologous serum skin test were positive in approximately 60% of the sample, reinforcing their supportive, though not definitive diagnostic value. In summary, APD is an important differential diagnosis in female patients with dermatitis and should be considered in clinical investigations.

## Data Availability

The research data are described in the article presented.
